# Richard Dean: The Value of Humanity in Kant’s Moral Theory: Clarendon Press, Oxford, 2006, pp. x + 267. Cloth, £28.12

**DOI:** 10.1007/s11019-019-09926-2

**Published:** 2019-09-30

**Authors:** Victor Chidi Wolemonwu

**Affiliations:** Department of Philosophy, University of Sheffield, Sheffield, United Kingdom

**Keywords:** Richard Dean, Immanuel Kant, Autonomy, Informed consent, Dignity, Duty

## Abstract

This is critical review of Richard Dean’ book, The Value of Humanity in Kant’s Moral Theory. Dean’s book was evaluated, and some of his interpretations of Kant were critiqued. However, it concludes that Dean’s book is illuminating especially, as regards the distinction he made between consent and informed consent and their roles in biomedical practice.

*The Value of Humanity in Kant’s Moral Philosophy* is a critical exploration of the core themes in Kant’s ethics, namely, the notions of humanity, dignity, good will, autonomy and end-in-itself. There is no gainsaying that Kant’s humanity thesis, which is the bedrock for which this book was written, appears to stand out among the other two formulations—The Universal Law of Nature Formulation and the Autonomy Formulation. Most commentators seem to allude to this fact in that the humanity thesis seems logically attractive and appeals to our intuitions, and it is the grounds for all normative theories, especially, in applied ethics. As Richard Dean points out at the start of this book, the tricky challenge of the humanity thesis is the explanation of the moral obligations it implies (p. 4). Addressing this challenge, therefore, demands an apt explication of its logic vis-à-vis our conventional moral intuitions. This, among other things, is a task that Dean sets to achieve in this book.

Dean begins his analysis with a distinction between the meaning of humanity as construed by some contemporary interpreters and the actual meaning of humanity. According to him, “specialists in Kant’s ethics regard Kantian humanity as some feature possessed by rational beings, and not just as the property of being a member of the human species. This seems correct to me. But there is more disagreement than is generally recognized about exactly which characteristic of rational beings Kant means to pick out as the ‘humanity’ that must be treated as an end in itself” (p. 5). The disagreement is because these scholars believe that the status of humanity is accorded to all well-informed adult human beings that possess at least a minimal rationality, which is logically erroneous. “Humanity, in the sense of the humanity formulation, is indeed equivalent to some feature possessed by rational beings, but not by all minimally rational beings. More so, ‘humanity’ is Kant’s name for the more fully rational nature that is only possessed by a being who accepts moral principles as providing sufficient reasons for action. The humanity that should be treated as an end in itself is a properly ordered will, which gives priority to moral considerations over self-interest. To employ Kant’s terminology, the end in itself is a good will” (p. 6).

Consequently, it is not all human beings that are qualified to be enrolled into the college of Kantian humanity. The only qualification is that such human being, despite being rational, must give priority to moral considerations over personal inclinations and aggrandizements. This interpretation seems problematic on two grounds. First, it seems to suggest that criminals may not be qualified to be accorded the status of humanity, and by extension, may not possess dignity, since their actions are influenced by their inclinations and appeal to the hypothetical imperative. A closer reading of Kant seems to contradict this Deanian interpretation. Because, Kant equates dignity with humanity, and believes that the possession of dignity and being part of humanity is not limited to acting in accordance to good will. Moreover, to possess dignity does not imply acting in a morally good or plausible way. Dignity is a definitive quality that defines the nature of human beings, and it is, according to Kant, a prerogative, which human beings have over all other natural beings (*Groundwork*, p. 4:436, p. 4:438). The implication of this is that possessing dignity is independent of our moral probity.

The second problem is the restriction of the idea of humanity and dignity to only those who are rationally competent, and the preclusion of marginal cases like little babies, demented patients and those in coma. This interpretation seems anti-Kantian. Kant views dignity as an inherent moral worth, which defines the humanity (humanness) of *all* human beings. The definition of humanity as a status for ‘fully rational nature that is only possessed by a being who accepts moral principles as providing sufficient reasons for action’, therefore, seems problematic. In his *Metaphysics of Morals*, Kant believes that what characterizes *humanity* and distinguishes it from *animality* is the *capacity* to set ends—‘any end whatsoever’ (p. 392). Being able to set ends isn’t an exclusive privilege a of *fully* rational nature but a privilege of *all* rational nature, irrespective of their degree of maturity. Children or demented patients may not be able to set ends in an instrumental or practical fashion given some physiological and psychological inhibitions, that does not suggest that they lack the capacity to set ends. For those with severe neurological or biological disabilities, they remain perpetually inhibited. So, we could say that their capacity to set ends is inert or latent.

Dean attacks the interpretation of the notion of good will, especially as defined by H. J. Paton and Lewis White Beck. According to him, Paton and Beck view good will as “’… one which acts for the sake of duty…. An action having this motive (the motive of duty) is moral…’. According to this view, a good will is exactly a will that performs dutiful actions because they are required by duty” (p. 19). Dean believes that it is plausible to argue that an agent possesses a good will if his actions are done from the motives of duty, the will is not a passive possessor but an active possessor of that which is good. The fundamental characteristic of human is the power of choice. A good will, therefore, is one that chooses to perform a morally worthy action. The idea of good will, thus, according to Paton and Beck seem oversimplified.

Dean distinguished between two kinds of will: will as choice and the will that makes available the practical laws upon which all choices are based. Whereas the former is referred to as *Willkur*, latter is referred to as *Wille*. The morality worthiness of an action depends on the extent to which *Willkur* conforms with *Wille*. When the *Wille* of an agent governs his *Willkur*, we say that such moral agent, aside being fully rational, possesses a will that is good. “In order for an agent’s will to be good, she must have a commitment to act morally even when acting morally requires her to forgo the satisfaction of inclinations (she must make the moral law ‘the supreme condition of the satisfaction’ of her inclinations)” (p. 20). This Deanian interpretation seems to give credence to Marcia Baron’s idea of over-determinism, in which duty regulates our inclinations as a secondary motive, without extricating it from the morally active scene. Agents control their inclinations, making sure that for no reason would their inclinations become the determining factor of his moral action. This act of control is a moral commitment, which an agent must develop and master in order to perform actions that can be adjudged morally justified.

Another concept which Dean thinks is integral to Kant’s moral theory is the concept of autonomy. Autonomy is the underlying principle of all human dignity and it is a defining principle of all human nature. It is reason that makes autonomy possible. Dean lists three functions of reason in Kant’s Philosophy: “…reason is pure self-activity, which makes rational beings fundamentally active instead of passive. In his theoretical philosophy, reason is the faculty that spontaneously and freely provides rules for organizing the ‘intuitions’ of sense that we passively receive. In his practical philosophy, reason freely and spontaneously provides the moral principles that present truly self-given reasons for action, independent of the influence of inclinations (p. 227). To be autonomous, hence, is to be a self-legislating agent, and by extension an end-in-itself. This sort of value-laden role ascribed to autonomy stirs an ontological conflict between the humanity formulation and the autonomy formulation. Which of these formulations underlies morality? Dean explains that based on Kant’s analysis, autonomy is the foundation of dignity. To wit, the possibility of a good will depends on whether moral principles that guide our actions are autonomously legislated. Without this autonomous legislation, the actions of human beings may be based on ‘their strongest desire’. And, their actions would not be different from other beings in nature (p. 228). Moreover, viewing autonomy as an end-in-itself does not imply overriding the strategic role of good will in the moral scheme. Autonomy and good will complements each other. They are two sides of a moral coin. While a good will is the prerequisite that renders the contingent moral aspirations of an agent morally valuable, the will itself, must be autonomous in order to choose what is good. The will must autonomously legislate moral principles that are not subject to any inclination but are only constrained by moral laws.

Since the postulation of the notion of autonomy by Kant, some bioethicists like Gerald Dworkin have appropriated the concept as a normative principle to evaluative doctors-patients relationship (1976, p. 23). The concept of autonomy also underlies most biomedical research guidelines, and it accentuates the ethical imperative that all potential research participants “have a right to choose freely whether to participate in research” (CIOMS 2016, p. 34), because they are intrinsically valuable and morally autonomous. Historically speaking, the idea of autonomy is often invoked as a reaction against the culture of paternalistic decisions, which underlie the medical profession for several decades, especially, in America and several parts of Europe (even though it is significantly present in medical practices in Africa). The wrongness of paternalism, as some anti-paternalistic proponents have averred, is that it interferes with the autonomous decisions and actions of individuals (Birks 2018, p. 138). The respect of autonomy seems to imply the respect of an individual’s right to self-determine their actions and legislate on their preferred choices, without any interference, whatsoever. However, Dean contends that “… respect for autonomy amounts to little more than a demand to let patients make choices about their medical care or for potential volunteers to either accept or refuse research participation. This emphasis on choice, sometimes to the exclusion of any other aspect of respect for autonomy, is understandable, given the original role of the principle” (p. 199). The emphasis on the rights of patient’s autonomy, thus, is to show what could be wrong when patients are excluded in medical decision making about their medical treatment.

The demand that patients’ choices be respected may be morally acceptable, nevertheless, it creates a leeway for blind consent, which medical experts often exploit. Therefore, the respect for autonomy requires a higher demand for rationale behind all possible alternatives opened to choice. The justification for requiring a doctor to make adequate information available before obtaining consents from patients to this end, according to Dean, “allows patients to make choices that fit with their overall desires, goals, and attitudes. This is the real requirement and intuitive force of the principle of respect for autonomy, and it accords with most explicit formulations of that principle” (p. 200). Dean’s introduction of the notions of choice and autonomy gives an illuminating insight into consent and informed consent dichotomy, and their diverse role in biomedical practice. While the idea of consent creates a possibility of blind choice, informed consent opens a more robust ethical requirement, whereby the doctor becomes obligated to provide accurate information which would aid the participant or patient to make choices that express their desires and aspirations as self-legislating agents, and also, refrain from interferences that are gratuitous (p. 203). Importantly, informed consent bids the physician to make judgements that would be for the patient’s best interests. The judgments of physicians or biomedical researchers, therefore, ought to incorporate *the duty of beneficence* (obligation to improve the health and well-being of patients or research subjects and *non*-*maleficence* (the obligation to ensure a non-deliberate cause of harm).

The idea of individual autonomy represented by Gerald Dworkin and also reflected in Biomedical Research Ethics guidelines have come under attack because of its over-burdened emphasis on the unrestricted right of an individual to make decisions based on motives, desires and aspirations, and in protection of their interests and well-being. Childress, for instance, contends that the problem of individual autonomy is that it focuses on just one aspect of our personhood, which is, self-determination, neglecting the socio-cultural and historical embodiment of our personhood. Also, the proponents of individual autonomy do not consider the possibility of misplaced, ambivalent, or even contradictory preferences (1990, p. 13). In order cases, some choices may be borne out the motivation to impress the consentee. For instance, if a well-known clinical researcher goes to the locality where she is well-known to recruit research participants, the possibility of recruiting several participants is very high. But, irrespective of the informed consent rituals, the participants may decide to consent based on the grounds of deference to the researcher, even when they would prefer to choose not to participate when it involves someone else. Childress, and of course, Beauchamp believe that a better idea of autonomy should be personal autonomy or what Dean calls ‘a Minimalist Self-Deterministic account of Autonomy’ (MSDA). According to them, personal autonomy or MSDA entails the capacity to make indivdual decision or choose a preferred course of action, which at minimum, is free from controlling influence by others and from limitations, such as insufficient understanding that makes it impossible for the autonomous agent to make meaningful choices (Beauchamp and Childress 2001 qtd in Varelius 2006, p. 377).

A significant criterion of personal autonomy as construed by Beauchamp and Childress is that in as much as an individual possesses a moral capacity to make choices and decisions, such capacity must not impede certain factors that could regulate the decisions he makes (1994, p. 121). Where your choice poses a threat to your attaining desired goals, other factors external to you must be allowed to act on your choice so that the desired goal can be attained. A fundamental consideration in ascertaining moral grounds for medical decision should be based on medical interest. For instance, in the case of a mentally retarded person, “it is irrelevant in determining whether treatment is in the patient’s best interest” (1994, p. 216). There should not be confusion between the value of life for others and the patient’s quality of life. In a situation where a patient is incapacitated and incompetent to make decisions as regards his or her health condition, the incompetent patient’s best medical interests generally should suffice even if these interests’ conflict with the interests of the family of the incompetent patient. According to Dean, the choice of taking a minimalist stance on autonomy is to make “autonomous choice the central object of concern in the principle of respect for autonomy, instead of assigning this central role to autonomy as a property of persons” (p. 204). Nevertheless, this stance of autonomy is problematic because though it permits a minimal interference on an agent’s choice if their choice were incompetently made, it restricts interference on “the inconsistent decisions of someone halfway in the grip of unusual religious beliefs” (p. 204) like an individual who refuses to accept blood transfusion after childbirth, even when it is clear that not doing so would result in her death. More so, if the autonomous and intentional choice is what MSDA seems to promote and preserve, Dean believes that it is difficult to see why all choices shouldn’t be respected, even if it is unreflective. This is not to suggest that Dean supports unreflective choices but to point to the inadequacies of individual and personal accounts of autonomy.

Kant’s view of autonomy, which is distinct from individual and personal autonomy is ‘metaphysically laden’ and morally grounded. It is metaphysically laden because he dissociates autonomy from the individual and the personality of the individual and anchors it on the will. It is therefore the will that is autonomous. Kant explains that a will that is autonomous is that which conforms to the moral law. The will is a kind of causality, which defines all beings that are part of rational nature. To be human, thus, is to possess a will. The nature of a will depends on the motivations that stimulates its willing. A good will, thus, “is not good because of what it effects or accomplishes, because of its fitness to attain some proposed end, but only because of its *volition*, that is, it is good in itself and, regarded for itself, is to be valued incomparably higher than all that could merely be brought about by it in favor of some inclination and indeed, if you will, of the sum of all inclinations” (*Groundworks*, p. 4:395 Emphasis is mine). Dean explains that because good will is *volition*-*dependent*, it is the grounds for autonomy. According to him, to be autonomous is to possess good will. In other words, to be able to perform an autonomous action, one must necessarily possess a good will, and it is what gives value to the action of a moral agent (p. 255). To act on good will is to act morally, but to act without recourse to good will is “to choose to act immorally in order to satisfy inclinations” (p. 255). So, to perform good or morally acceptable acts is to act in accordance to autonomy. It also means to act on the grounds or motivation of reason because only reason and not incentive directs an act to conform to moral law. This idea of autonomy is restricted to an agent that wills a moral action. It is not relational—it is not expressed in relation to another moral agent.

How would Deanian-Kantian good will interpretation of autonomy address the problems of informed consent and moral decision-making in medical practice and biomedical research? A possible response by Dean would be that a doctor or a patient, for instance, could be said to have acted autonomously if their actions are morally grounded, that is, if their actions are morally justified. If a doctor gives a false diagnosis to a patient in order to keep them perpetually and hopelessly in their hospital just for commercial gains, such a doctor acts immorally and cannot claim to have acted autonomously as the motivation of the action isn’t good will but self-aggrandizement. While this may seem like an abstract moralization, it does not seem to address in practical terms some complexities that occur in medical practice. For instance, it doesn’t answer the question of whether a doctor acts autonomously when he treats a cancer patient or performs a Cesarean Section on a woman with an ectopic pregnancy against the will of the patient. It is also difficult to see how the good will account of autonomy helps to explain patients or research participants’ decisions. For instance, it is difficult to see how that account holds a clinical researcher culpable for recruiting a participant who agreed to participate in a clinical research on the grounds of (monetary or therapeutic) incentives advertised during the recruitment process. If a participant willingly agrees to participate in such a trial not through coercion but simply to enjoy the gains of the advertised incentives (either because they are sick or due to their impoverished conditions), can we say that they acted autonomously? Or, if a cancer patient agrees to the decision of doctor to treat her without being interested in the details, can we say that the cancer patient acted autonomously? The good will account does not offer any response.

The abstract moralization of autonomy pokes Secker to claim that “Kant’s concept of moral autonomy is not a concept we should attempt to “plug into” bioethics’ principle of respect for autonomy because of its undesirability as an ideal and its impracticability in assessment contexts” (1999, p. 52). Endorsing Secker’s attack on this Deanian-Kantian abstract moralization of autonomy, Saad argues that

it is unrealistic because many people, especially sick and vulnerable patients, are incapable of the sort of philosophical gymnastics Kant requires. Furthermore, because Kant equates acting autonomously with acting morally, applying Kant’s autonomy to clinical practice would, in a sense, make doctors arbitrators of patient morality. If patients have a duty to act autonomously… and if doctors are to gauge patient autonomy, the patient will be subjected to a physical and moral examination before undergoing treatment (2018, p. 130).

All these criticisms are plausible only because Kant’s view of autonomy is explored in part.

A broader and practical exploration of Kant’s view of autonomy reveals that while Kant is interested in moral goodness of an agent, he is also interested in how that morally good agent treats another agent. For instance, Kant believes that it is morally despicable to tell lies. So, if a doctor lies to a cancer patient that there is no hope for them to get healed just to make the patient stay perpetually in their care simply for commercial gains, Kant believes that such action is morally despicable because “whatever militates against frankness lowers the dignity of man” (*Lecture on Ethics*^1963^, p. 231). Kant defines a lie as a “*falsiloquim in praejudicium humanitatis* - false statement prejudicial to humanity” (*Lecture on Ethics*^1963^, p. 227, translation is mine). He however adds that not every statement that is untrue is a lie. Because “if we were to be at all times punctiliously truthful, we might often become victims of the wickedness of others who were ready to abuse our truthfulness” (Lecture on Ethics, p. 228). A pharmacist who sells vitamin C to a drug addict in place of Codeine tablet may have deceived the drug addict to believe that they have bought a psychoactive drug when they have only got a medicine that would help boost their body vitamin. Kant does not believe that the pharmacist has acted in a morally despicable way but in good will. But Dean’s good will account does not account for the role lies or truthfulness plays in defining moral goodness.

The concept, which is very central to Kant’s moral autonomy, which is missing in the good will account is the idea of *mere means*. To be autonomous is to act in such a way that one does not treat the other as a mere means. The ‘non-use as mere means’ view suggests that all human beings ought to be treated with respect because they possess intrinsic value. Though Dean situates *valueness* in the moral goodness of an action, Kant believes that irrespective of our moral status, because we are part of rational nature, we are intrinsically and unconditionally invaluable, that is, we possess inviolable dignity. For Kant, “a human being… is not a *thing* and hence not *something* that can be used merely as a means but must *in all his actions* always be regarded as an end in itself” (*Groundwork*^1997^, p. 38). To act autonomously is to treat others as *ends* rather than as *mere means*. A will which makes it possible for an agent to treat others as ends is a good will. A good will is a will that conforms to the moral principles of reason. Another way to explain this is to say that an individual performs actions autonomously when they will action that are determined by rational moral principles. An autonomous action is distinguished from a heteronomous action. While autonomous actions are motivated by practical reason, heteronomous actions are motivated by feelings, inclinations ad pathologies. One cannot claim to be autonomous when their actions are controlled by feelings and inclination because the reason for such actions is illegitimate as well as defective. A captivating way of describing an autonomously willed action is to see it as “the product of a process of deliberation which involves *scrutiny of the reasons* underlying our proposed actions to ensure that they meet certain standards of rationality” (Campbell 2017, p. 388). So, scrutinizing our actions in conformity to moral imperatives is vital to autonomy. More specifically, our autonomous will is constrained by moral principled to act in the morally plausible way. This broader account of autonomy views autonomy not as a self-deterministic capacity, which is devoid of interference. This account is based on the capacity of a will to will a good action through rational deliberation. Deanian good will account of autonomy does not consider this aspect of moral scrutiny or deliberation. But a broader Kantian reading shows that this is very fundamental in Kant’s discussion of autonomy.

There is, therefore, a sense in which Kant’s moral autonomy sits prominently within an ineluctable pluralistic moral deliberation that is primal to bioethics and medical practice. Kant’s autonomy, just to reiterate, is manifested in a moral action in which moral duties or obligations are met, and in which the rights, well-beings and dignity of others are respected. This is because, an individual cannot claim to be autonomous when they don’t act in reference to every other moral agent. So, every human action ought to base on “informed reason and autonomously held, principled commitments” (Stirrat and Gill 2005, p. 127). More specifically, Kant’s view of autonomy “requires that we act only on principles that can be principles for all; it provides a basis for an account of the underlying principles of universal obligations and rights that can structure relationships between agents” (O’Neill 2002, p. 96). So, autonomy that is based on the ‘underlying principles of universal obligations’ does not tolerate deception, manipulation or coercion. This is expressed through “truthful communication, through care not to mislead, through avoidance of exaggeration, through simplicity and explicitness, through honesty in dealing with others, in a word, through trustworthiness” (O’Neill 2002, p. 98). In the field of bioethics and medical practice, Kant’s autonomy could be expressed, thus, where in a clinical researcher and a research participant, for instance, communicates to each other truthfully and attempts not to mislead each other. In the case of complex moral deliberation, the deliberating agents must act in a way that stimulates or promotes trustworthiness. If, for instance, a patient wishes to accept blood transfusion but is constrained by some religious belief system, and the doctor believes that is the only way to save her from an impeding danger of possible death, it is morally instructive that whatever decision that is arrived at must be such that is morally motivated by honesty, trustfulness and care not to mislead.

Even though the good will account expressed by Dean is insufficient to explain how Kant’s view of autonomy could help to address pluralistic moral issues that arise in bioethics and medical practice, it is, however, necessary. But, to see how that account could be applied to bioethics and medical practice, it must to be read in conjunction to the mere means principle, in which our obligations to act are informed vis-à-vis respect for other moral agents (whether rationally competent or not).
